# Chemometric approach to characterization of the selected grape seed oils based on their fatty acids composition and FTIR spectroscopy

**DOI:** 10.1038/s41598-021-98763-6

**Published:** 2021-09-28

**Authors:** Mašán Vladimír, Arkadiusz P. Matwijczuk, Agnieszka Niemczynowicz, Radosław A. Kycia, Dariusz Karcz, Bożena Gładyszewska, Lidia Ślusarczyk, Patrik Burg

**Affiliations:** 1 Department of Horticultural Machinery, Mendel University in Brno, Faculty of Horticulture, 691 44 Lednice, Czech Republic; 2 Department of Biophysics, University of Life Sciences in Lublin, 20-950 Lublin, Poland; 3 University of Warmia and Mazury in Olsztyn, Faculty of Mathematics and Computer Science, 10-710 Olsztyn, Poland; 4 Cracow University of Technology, Faculty of Materials Engineering and Physics, 31-155 Krakow, Poland; 5 Masaryk University, Faculty of Science, Kotlářská 2, 602 00 Brno-střed, Czechia; 6 Department of Chemical Technology and Environmental Analytics (C1), Cracow University of Technology, Faculty of Chemical Engineering and Technology, 31-155 Krakow, Poland

**Keywords:** Biotechnology, Plant sciences, Environmental sciences, Mathematics and computing, Physics

## Abstract

Addressing the issues arising from the production and trade of low-quality foods necessitates developing new quality control methods. Cooking oils, especially those produced from the grape seeds, are an example of food products that often suffer from questionable quality due to various adulterations and low-quality fruits used for their production. Among many methods allowing for fast and efficient food quality control, the combination of experimental and advanced mathematical approaches seems most reliable. In this work a method for grape seed oils compositional characterization based on the infrared (FTIR) spectroscopy and fatty acids profile is reported. Also, the relevant parameters of oils are characterized using a combination of standard techniques such as the Principal Component Analysis, k-Means, and Gaussian Mixture Model (GMM) fitting parameters. Two different approaches to perform unsupervised clustering using GMM were investigated. The first approach relies on the profile of fatty acids, while the second is FT-IR spectroscopy-based. The GMM fitting parameters in both approaches were compared. The results obtained from both approaches are consistent and complementary and provide the tools to address the characterization and clustering issues in grape seed oils.

## Introduction

Globally, there are more than 120 million tons of edible oils and fats produced, of which approximately 80% are derived from various plant sources and thus referred to as vegetable oils^1^. Typical commercially available plant oils are colza, pumpkin, olive, sunflower and others. In terms of application on a larger scale, some new possibilities are offered by grape seed oil^2^. The raw material for its production is vine seeds, which are obtained from pomace, a waste product from the processing of grapes in the winery and makes up 20% (v/w) of the total amount of processed raw material. Pomace is traditionally considered an economic and environmental problem. It is now becoming increasingly recognized as a valuable commodity for the production of added value products^3^. Oil from grape seeds is an attractive raw material in the food industry for its dietary value as well as a substance for cosmetic and pharmaceutical applications^4,5^. It has a high content of essential fatty acids and tocopherols^6^. The production of these oils in the Czech Republic and abroad has been growing significantly and it opens up new possibilities for vineyard and winery operations in the application of the residual primary products^7^.

According to the chemical composition, grape seed oil falls into the category of oils with a high content of unsaturated fatty acids^8^. On average, grape seed oil is 90% composed of poly- and monounsaturated fatty acids, which are responsible for its value as nutritive edible oil. In particular, linoleic acid (58–78%, 18:2n − 6), oleic acid (3–15%, 18:1n − 9), and minor amount of saturated fatty acids (10%) are its main constituents. Unrefined oils contain bioactive compounds, including tocopherols (5–52 mg/100 g) and numerous phenolic components, consisting of low and high molecular plant phenolics, which may be responsible for various beneficial effects demonstrated by vegetable oils^9,10^. However, all the positive effects manifest primarily in oils of specific purity, especially those that are unadulterated and maintain their time of consumption^11,12^. One of the first use of Fourier Transform Infrared Spectroscopy (FTIR) for analysis of grapes and wine started with near-infrared spectroscopy^13,14^. Due to higher accuracy and more constituents and properties which can be quantified, the analysis of grapes and wine is nowadays based mainly on FT-IR spectroscopy combined with advanced statistical methods (also known as chemometrics analysis)^15^ and more frequently with the use of machine learning methods^11,16^. During the last decades the FTIR spectroscopy combined with the advanced statistics mentioned are increasingly used for extended studies on grapes and wines, and usually effects in excellent precision and accuracy of results obtained. These techniques are fast and reproducible for identifying the authenticity and adulteration of the wide variety of food and beverage products. The FTIR spectra coupled with statistical tools were used to evidentiate saccharides, alcohols, or other quality parameters. Among many qualitative chemometric methods, the Principal Component Analysis (PCA) is most often used. Moreover, there are many research works concerning authenticity and traceability, related to origin^17–19^.

Therefore in this work a various grape seed oils were compared with the use of chemometrics methods. Two different approaches namely the fatty acid profile-based and the FT-IR-based approach were applied. The strategy involved a combination of two clustering methods, namely the K-Means and Gaussian Mixture Model (GMM). Two-dimensional feature space resulting from a dimension reduction by Principal Component Analysis (PCA) was used in GMM for clustering various grapevine oils.

## Results and discussion

### Fatty acid compounds of the selected grape seed oils

The fatty acid composition of the oils extracted from eight grape cultivars and 2 years of harvesting is shown in Table 1 linoleic (70.10–71.55%), oleic (15.33–17.28%), and palmitic (6.84–8.18%) acids were the predominant fatty acids in oils, consistent with previously reported data^8,20^. The differences between the selected acids compared to varieties and vintages are given in %-age units.

**Table 1:** The relative concentration of fatty acids in grape oils.

Oils	Concentration of fatty acids in the grape oils (%)								SFA (%)	MUFA (%)	PUFA (%)	ρ (g/ml)	μ (mPa s)
	C16:0	C16:1n7	C18:0	C18:1n9c	C18:2n6c	C18:3n3	C20:1	Others					
Dornfelder 2015	8.18	0.16	3.16	15.61	71.05	0.55	0.20	1.09	11.40	16.77	71.84	0.947	61.03
Páláva 2015	7.67	0.15	3.62	16.17	70.66	0.41	0.17	1.15	11.37	17.30	71.32	0.944	60.31
Riesling 2015	6.87	0.19	3.87	17.14	70.15	0.45	0.18	1.15	10.79	18.33	70.88	0.939	59.64
Pinot Gris 2015	7.45	0.15	3.76	15.33	71.55	0.42	0.21	1.13	11.27	16.45	72.27	0.907	60.84
Zweigeltrebe 2015	7.33	0.12	3.88	16.84	70.18	0.38	0.19	1.08	11.27	17.92	70.82	0.931	61.04
Tramin 2015	6.87	0.17	3.74	17.16	70.25	0.42	0.20	1.19	10.69	18.36	70.95	0.940	59.12
Hibernal 2017	6.92	0.17	3.87	17.14	70.10	0.42	0.20	1.18	10.87	18.34	70.79	0.942	59.77
Sauvignon 2017	6.84	0.17	3.79	17.28	70.12	0.45	0.21	1.14	10.70	18.45	70.85	0.939	59.96
Zweigeltrebe 2017	7.41	0.13	3.91	16.29	70.59	0.43	0.18	1.06	11.38	17.33	71.29	0.935	61.2
Neuburger 2017	7.83	0.15	3.54	16.70	70.20	0.39	0.16	1.03	11.47	17.69	70.84	0.944	59.01

The concentration of saturated, monosaturated, and polyunsaturated are presented along with the physical properties (measured at 20 °C), which are the apparent viscosity (µ) and the mass density (ρ).

### Chemometric analysis of fatty acid compounds and physical parameters of selected grape seed oils

#### Correlation analysis

Analysis of correlations between unsaturation and the physical parameters was the first step in characterizing selected grape seed oils. Therefore, the fatty acids were grouped into saturated fatty acid (SFA), monosaturated fatty acid (MUFA), and polyunsaturated fatty acid (PUFA). The relationship between the number of unsaturation and the physical parameters was obtained by analyzing correlations between concentration of SFA, MUFA, PUFA, and the values of physical parameters: mass density and apparent viscosity. Pearson’s correlation coefficients were presented in Table 2. A high value of modulus of coefficients between two considered variables explains the direction of their relation.

**Table 2:**
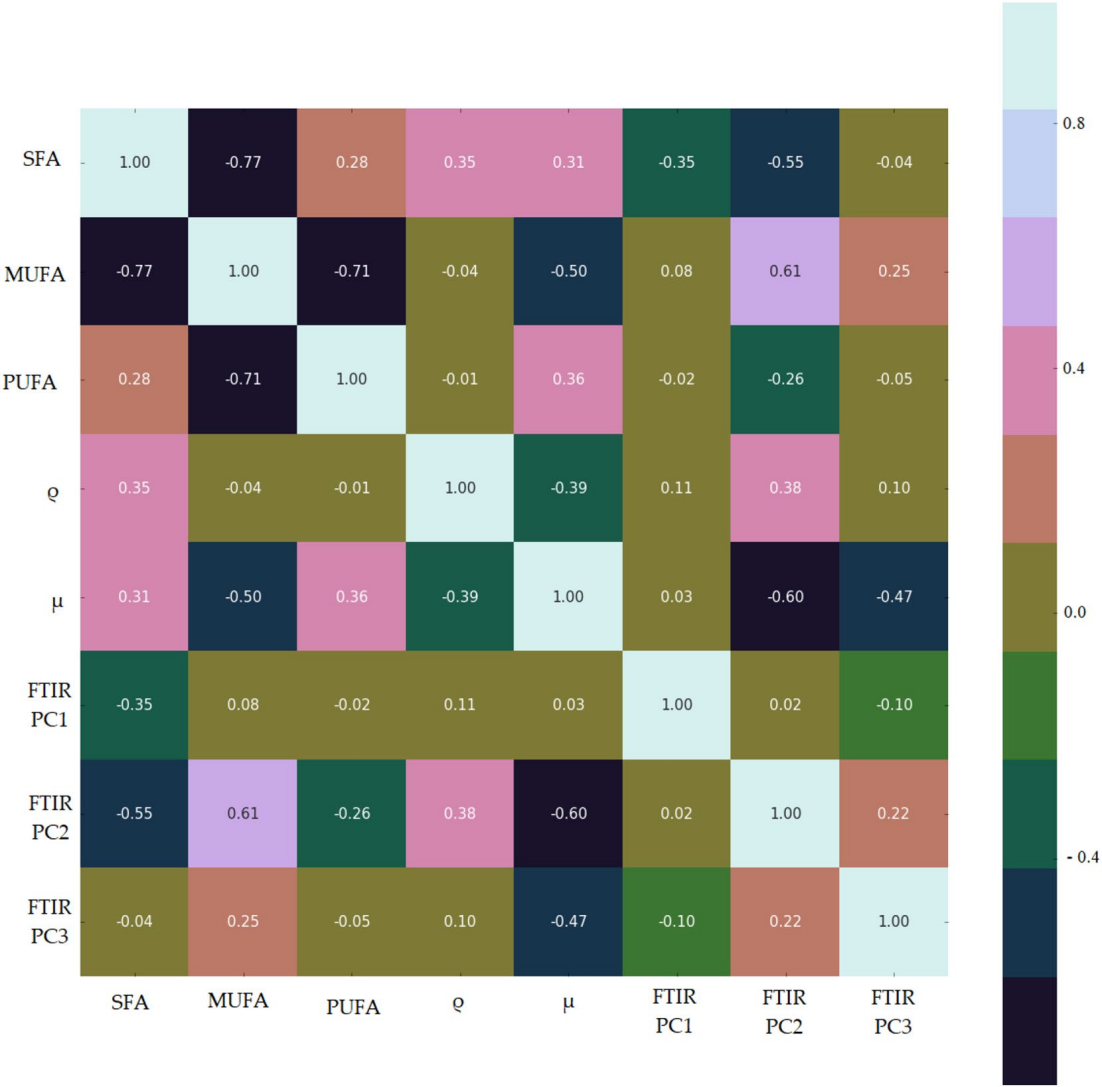
Correlation coefficients between the relative concentration of SFA, MUFA, PUFA physical properties, and PC2, PC2, and PC3 indexes.

The SFA concentration in analyzed oils has significant correlations with MUFA (|R| = 0.77) and PC2 (|R| = 0.55), but small values of correlations coefficients with other variables (|R| < 0.5). A high correlation relations were observed between MUFA and PUFA (|R| = 0.71), MUFA and µ (|R| = 0.5), MUFA and PC2 (|R| = 0.61). The difference between concentrations of SFA and MUFA in analyzed oils is lower than the difference between SFA and PUFA. These relations are shown in correlation analysis. PC1 and PC3 present lower values of correlation coefficients when compared to PC2.

#### PCA

In the first approach for characterization of grape seed oils the PCA was applied to analyze seven common fatty acids, three groups of fatty acids and two physical parameters to obtain a linear estimate of dimensionality. Based on the Kaiser criterion in PCA, three components having eigenvalue higher than 1 were determined. The first three main components explained 88.66% of the total variance, and two components explained above 70% of it. Therefore, to simplify the description, we consider only the first two components in the following. The first component, PC1, explained 51.06%, the second PC2—19.2%, and the last, PC3-18.4%. The highest values of PC1 are for Pinot Gris 2015, Donfelder 2015, Paláva 2015, and Zweigeltrebe 2017. For PC2 dominant values are oils: Riesling 2015, Hibernal 2017, and Sauvignon 2017. According to the loadings the highest contribution of the fatty acid of PC1 take C18:2n6c and PUFA, while the mass density and C16:0 were for PC2.

#### k-Means for two PCs

**Figure 1:**
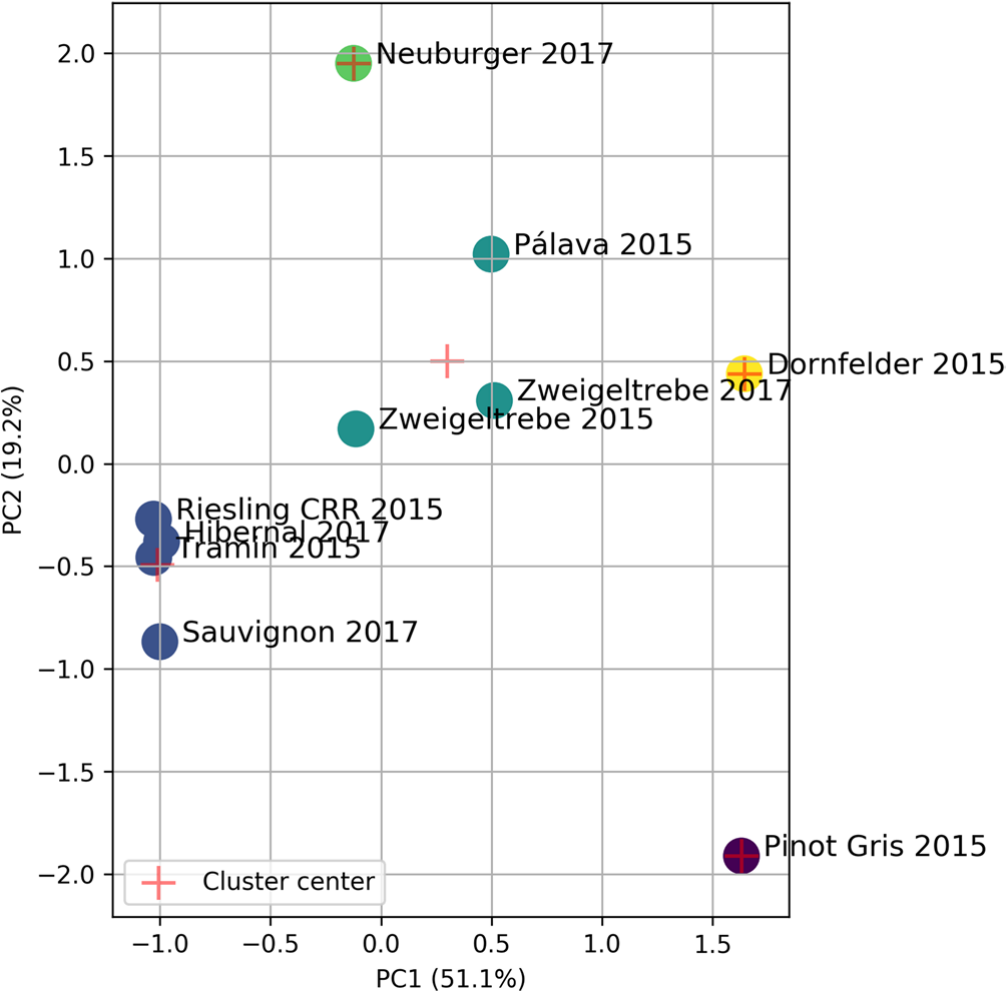
k-Means clusters for standardized PCs in analysis for fatty acid profile.

The second step involved the selection of initial values for the means in the mixture model. This was done by applying the k-Means method for normalized principal components, i.e., for reduced data set. As initial values the centers (or the means) of the clusters were taken. The sum of squared errors (SSE) suggests that the five clusters are an optimal choice. The clustering result is presented in Fig. 1.

#### GMM for clustering

**Figure 2:**
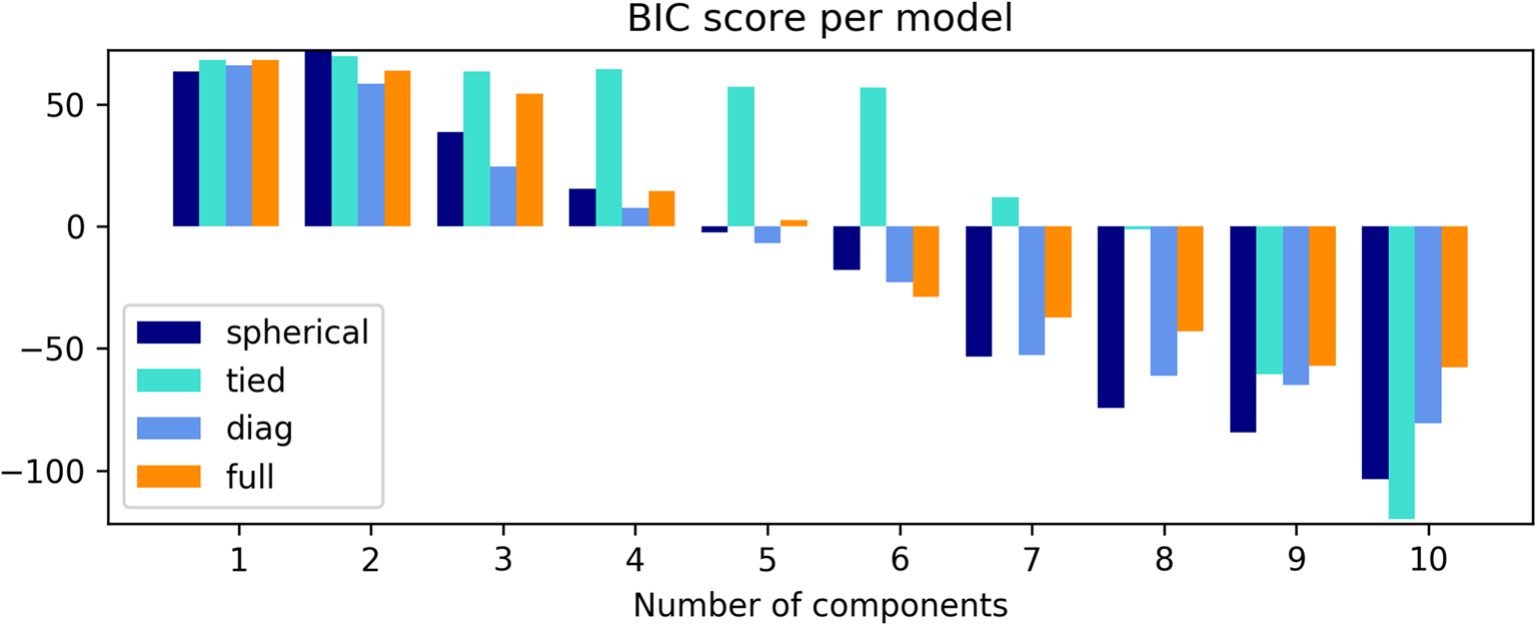
5 BIC score for various numbers of Gaussian components in GMM in analysis for fatty acid profile.

**Figure 3:**
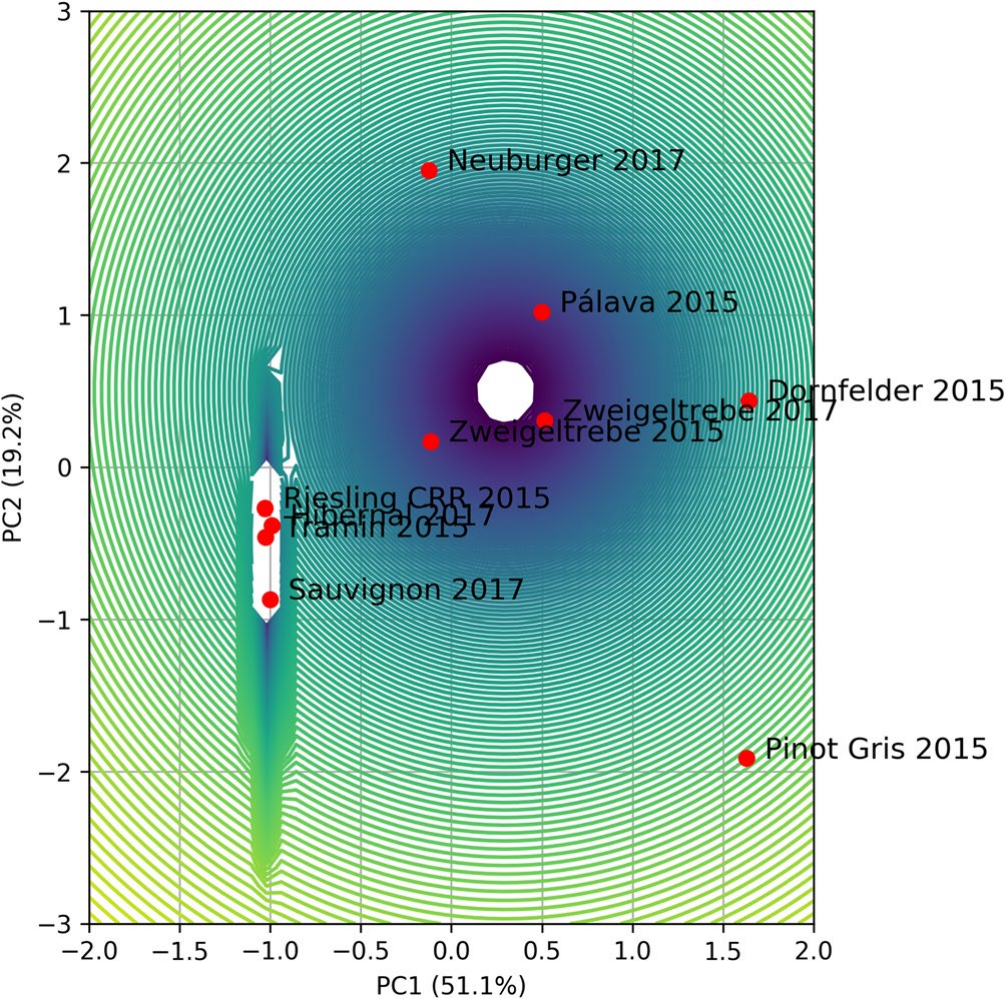
GMM for standardized 2PCs in analysis for fatty acid profile.

Next, the values for the parameters in GMM based on the number of clusters obtained with k-Means clustering were calculated. Five Gaussian components using Bayesian Information Criterion (BIC) were chosen in order to estimate the optimal model for Gaussians (Fig. 2), resulting in ‘diag’ (covariance matrix is diagonal) optimal Gaussian model for five components. The values of parameters from the fit are presented in Table 3, and the split into clusters is presented in Fig. 3.

**Table 3:** GMM parameters for standardized 2 PCs in analysis for the fatty acid profile.

Description	Values
Means	μ_1_ = [− 1.01211656 − 0.49377868]
	μ_2_ = [− 0.12413742 1.95111604]
	μ_3_ = [1.63061743 − 1.91205632]
	μ_4_ = [1.64397529 0.43813565]
	μ_5_ = [0.29933675 0.49930627]
Weights	π_1_ = 0.4
	π_2_ = 0.1
	π_3_ = 0.1
	π_4_ = 0.1
	π_5_ = 0.3
Covariance matrix (diagonal elements)	Σ_1_ = diag(2.65286297 × 10^−4^, 5.10190581 × 10^−2^)
	Σ_2_ = diag(1.0 × 10^−6^, 1.0 × 10^−6^)
	Σ_3_ = diag(1.0 × 10^−6^, 1.0 × 10^−6^)
	Σ_4_ = diag(1.0 × 10^−6^, 1.0 × 10^−6^)
	Σ_5_ = diag(8.52136779 × 10^−2^, 0.139321639)

#### FT-IR spectroscopy analysis

**Figure 4:**
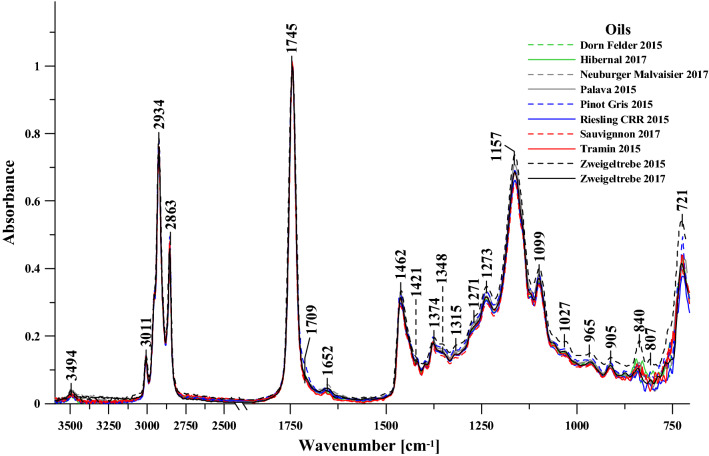
FT-IR spectra, normalised for the wavelength of 1745/cm, recorded for the respective grape seed oil samples: Dornfelder 2015 (dashed green), Hibernal 2017 (solid green), Neuburger 2017 (dashed gray), Pálava 2015 (solid gray), Pinot Gris 2015 (dashed blue), Riesling 2015 (solid blue), Sauvignon 2017 (dashed red), Tramin 2015 (solid red), Zweigeltrebe 2015 (dashed black) and Zweigeltrebe 2017 (solid black) respectively. All spectra are presented in the spectral range of 700–3600/cm and recorded at 23 °C.

The ATR-FTIR spectra for selected oil samples obtained from grape seeds of the selected cultivars harvested in the respective experimental years depending on the cultivar are presented in Fig. 4. The cultivars selected for the FTIR study included: Dornfelder 2015, Hibernal 2017, Neuburger 2017, Pálava 2015, Pinot Gris 2015, Riesling 2015, Sauvignon 2017, Tramin 2015, Zweigeltrebe 2015, and Zweigeltrebe 2017. For better convenience of analysis, discussion, and comparison of the respective samples studied, the spectra were normalized at the maximum of 1745/cm. The samples were placed on a ZnSe crystal and studied under N_2_ atmosphere (see “Materials and methods” section for details). Table 4 presents all the characteristic bands present in the oil samples selected for the study (from the first and second measurement years) from the aforementioned selected cultivars, and a correlation of the functional group vibrations with the corresponding bands (with a detailed literature review).

**Table 4:** The location of the maxima of the FTIR absorption bands, with the assignment of particular vibrations to the respective samples: Dornfelder 2015, Hibernal 2017, Neuburger 2017, Pálava 2015, Pinot Gris 2015, Riesling presents all the 2015, Sauvignon 2017, Tramin 2015, Zweigeltrebe 2015 and Zweigeltrebe 2017, registered within the spectral range of 700–3600/cm.

FTIR	Type and origin of vibrations
Positioning of band (/cm)	
3494	–C=O_w_ (overtone) and ν(=C–H_vw_, *trans-*)
3011	ν(=C–H_m_, *cis-*)
2934	ν_as_(–C–H_vst_, –CH_a_) and ν_s_(–C–H_vst_, –CH_a_) (aliphatic groups in triglycerides)
2863	
1745	ν(–C=O_vst_) in esters
1709	ν(–C=O_vw_) in acids
1652	ν_vw_(–C=C–, *cis-*)
1462	δ_vw_(–C–H) in CH_2_ and CH_3_ groups, deformation (scissor)
1421	ν_vw_(–C–H, *cis-*) deformation (wagging)
1374	ν_w, m, vw_(–C–H, –CH_3_) and deformation
1348	
1315	δ_m_(–C–H, –CH_3_)
1271	ν_m_(–C–O) or δ_m_(–CH_2_–)
1238	
1157	ν_st_(–C–O) or δ_st_(–CH_2_–)
1099	ν_m,vw_(–C–O)
1027	
965	δ_w_(–HC=CH–, *trans-*) out-of-plane deformation
905	
840	δ(–(CH_2_)_n_– and –HC=CH– (*cis-*) deformation (scissor)
807	
721	

*ν* stretching vibrations, *δ* deformation vibrations, *s* symmetric, *as* asymmetric, *st* strong, *w* weak.

It is worth noticing that all the infrared spectra (ATR-FTIR) of the selected oil samples, both in the first and the second year of the experiment, revealed highly intensive and distinct bands that could be correlated with specific functional groups vibrations originating from ingredients typically present in food products. A vast majority of edible plant fats, potential oily materials, are composed primarily of various fractions of triglycerides, differentiated mainly by the degree of unsaturation and the length of their respective hydrocarbon chains^21,22^. In many publications, the authors were able to match the particular bands present in the spectra of both animal and plant oils^21–31^ to specific vibrations of molecules or groups thereof. However, the majority of the literature available pertains to FTIR analyses of specific plants (e.g., rape) and animal oils, while only a few such studies have been carried out on the types of samples discussed in this work. Furthermore, a precise assignment of bands to a specific functional group is often problematic. Table 4 presents a detailed analysis of characteristic band frequencies with the most important widening observed in the oil spectra, and the correlations with their respective functional groups (including a review of relevant literature data^21,22,28–31^. Also, a subscript was used to account for the intensity of bands of the typical spectra in the infrared region. It is noteworthy that identifying stretching vibrations is significantly easier in this type of biological sample, especially when compared to deformation vibrations, which are often overlapped.

In the general characteristics of the selected oil samples spectra, vibrations of the methylene group located within the spectral range from 1350 to 1165/cm were observed^21,22^. In the case of our samples, these bands represented the stretching vibrations originating from the –C–H group bound to the –CH3 group (usually approx. 1350–60/cm, in our samples approx. 1348/cm) as well as deformation vibrations of the same group (present at approx. 1160/cm, in our case—1157/cm). It is noteworthy that the stretching vibrations of the (C–O) ester bond composed of two combined asymmetric vibrations are, in this case, vibrations of the C–C(=O)–O and O–C–C groups^31^. In the former case, the intensity of vibration is significantly higher^30^. The bands are present in the region from 1300 (as C–C(=O)–O, in our case approx. 1271/cm, as enhancement of the band with the maximum at approx. 1238/cm) and at approx. 1000/cm (in our case approx. 1027/cm for this group). In turn, the bands associated with saturated esters such as: C–C(=O)–O are found between 1240 and 1160/cm (in the case of the grapeseed oils samples selected for the study at approx. 1238/cm), while in the case of unsaturated, the vibrations usually emerge at lower frequencies^21^. At the same time, however, the O–C–O band often associated with primary alcohols is observed in the region from 1090 to 1020/cm (for the functional groups analyzed in our study, it was at approx. 1027/cm). In the case of secondary alcohols, the band usually emerges with the maximum at approx. 1100/cm (in our study approx. 1099/cm). Both types of esters described above are present in triglyceride molecules. However, authors often associate the band mentioned above (at approx. 1238/cm) exclusively with the out-of-plane bending vibrations of the methylene group^32^. The subsequent two bands presented in Table 4 (and in Fig. 4) have the maxima at approx. 1421 and 1315/cm, respectively (band widening, see Fig. 4, both for samples from the first and second measurement year). The first of said groups of vibrations (with the maximum at approx. 1421/cm) may originate from the vibrations of methyl groups in the aliphatic chains of the selected oil samples^21,32^. The second group of bands (i.e., the band widening) with the maximum at approx. 1315/cm (in all analyzed samples) was observed simultaneously with weak bands with maxima at approx. 965 and 905/cm. The 905/cm band present in all oil samples is associated with the stretching vibrations of cis-substituted olefinic groups^21^ and may also be associated with vibrations of the vinyl group.

The selected samples of grapeseed oil obtained in the two experimental years produced largely similar infrared spectra, but it should be noted that depending on the cultivar, certain differences were nonetheless observed that seem to be relatively characteristic and easily identifiable. Firstly, the studies revealed noticeably significant differences in terms of the respective bands’ intensity (not represented as the band levels were equalized at the peak related to the vibrations of the carbonyl group C=O to facilitate easier interpretation of the results), which seems to be related to the differences between the respective cultivars.

Another very characteristic region of vibrations contained bands with the maximum at approx. 1745/cm characteristic of stretching vibrations of the C=O carbonyl group^21^ in esters. Apart from the band characteristic for vibrations of the carbonyl group, on the lower wavenumber side there was also an enhancement with the maximum at approx. 1709/cm (distinctly less intensive in samples from, e.g., the Pinot Gris 2015 cultivar), which also corresponded to vibrations of the carbonyl group but occurred in the acid groups of the oil samples selected for the study^21,23,30^. The next band, with the maximum at 1652/cm corresponded to the stretching vibrations of the –C=C– group (from the *cis*-transformation)^21,28^. A characteristic region also contains vibrations with the maximum at 1462/cm originating from the deformation vibrations of the –C–H groups in –CH2 and –CH3 (bending vibrations). One should also mention vibrations in the region from 900 to 650/cm which represent characteristic deformation vibrations associated with the –HC=CH– groups (*cis*-conformation, out of plane) as well as the rocking vibrations of said groups ((–(CH2)n– and –HC=CH– (*cis*–))^21,28^.

As we proceed to vibrations in higher wavenumber regions, one should also mention the very significant stretching vibrations =C–H (*trans*-transformation) with the maximum at approx. 3066/cm (Table 4—very low intensity) originating from vibrations of the triglyceride fraction^21,33^ (in Fig. 4 with very low intensity—primarily in the Zweigeltrebe 2015 cultivar). In turn, the stretching vibrations of =C–H in the *cis*-configuration were observed as very characteristic and intensive vibrations with the maximum at approx. 3011/cm (Fig. 4 and Table 4). The vibrations with the maximum at approx. 2934, 2863/cm originate from the stretching –C–H vibrations in the –CH3, CH2 groups belonging to triglyceride aliphatic groups^21–29^.

It should also be noted that the spectra of the analyzed oil samples produced from the seeds of various grape cultivars (and from different years of the experiment) (Fig. 4) reveal noticeable differences in the shape of bands in the region from 1770 to 1660/cm. For most of the analyzed samples, one can clearly observe a slight band enhancement at 1745/cm (corresponding to the vibrations of the C=O, as already discussed above) on the lower wavenumber side, with a clear maximum at approx. 1709/cm^34^, which can also be correlated with forming a hydrogen bond between the C=O⋯H–O– groups (more intensive in the first year for the Pinot Gris 2015 group). Simultaneously with the emergence of the band at 1709/cm, we can observe a distinct change in the intensity of bands at approx. 1150–1070, 721/cm^28^, which can also be correlated to the stretching vibrations of C–O and C–C groups (described above). The bands, given the possibly decreasing affinity of the associated molecules with the formation of the C=O⋯H–O–H hydrogen bond, may suggest a slight increase in intensity thereof.

The spectral changes seem to correlate very well with the changes in the fatty acid profile presented in Table 1 and discussed in the first part of this section. Apart from the visible differences in the bands with the maxima at approx. 1710–1715, one should also emphasize the possibly most important observation, i.e., the emergence of a very clearly visible band with the maximum at approx. 840/cm (Fig. 4, Table 4) that may originate from the stretching vibrations on bonds existing between various acid fractions in the analyzed samples.

### Chemometric analysis of FTIR spectra of selected grape seed oils

#### PCA

According to the previously adopted procedure, firstly, the PCA method was applied to approximate the dimensionality of spectra data in a linear manner. Based on the Kaiser criterion in PCA three components having eigenvalue higher than 1 were determined. The first three main components explained 98.46% of the total variance, and two components explained above 95.18% of it. Therefore, we proceed further with our analysis using the first two components. The first component, PC1, explained 81.1%, the second PC2—14.09%, and the third, PC3, 3.27%. The highest values of PC1 takes for Zweigeltrebe 2017 and Neuburger 2017. In the case of PC2 dominant values are oils: Riesling 2015, Hibernal 2017, and Sauvignon 2017, which is a similar result like in analysis from fatty acid profile. According to the loadings, the highest contribution of FTIR spectra of PC1 take the vibration of w(–HC=CH–, *trans-*) out-of-plane deformation from the range 700-1500/cm, while for PC2 the vibration of (–C=O_vst_) in esters located in the region from 1600 to 2000/cm.

#### k-Means for two PCs

**Figure 5:**
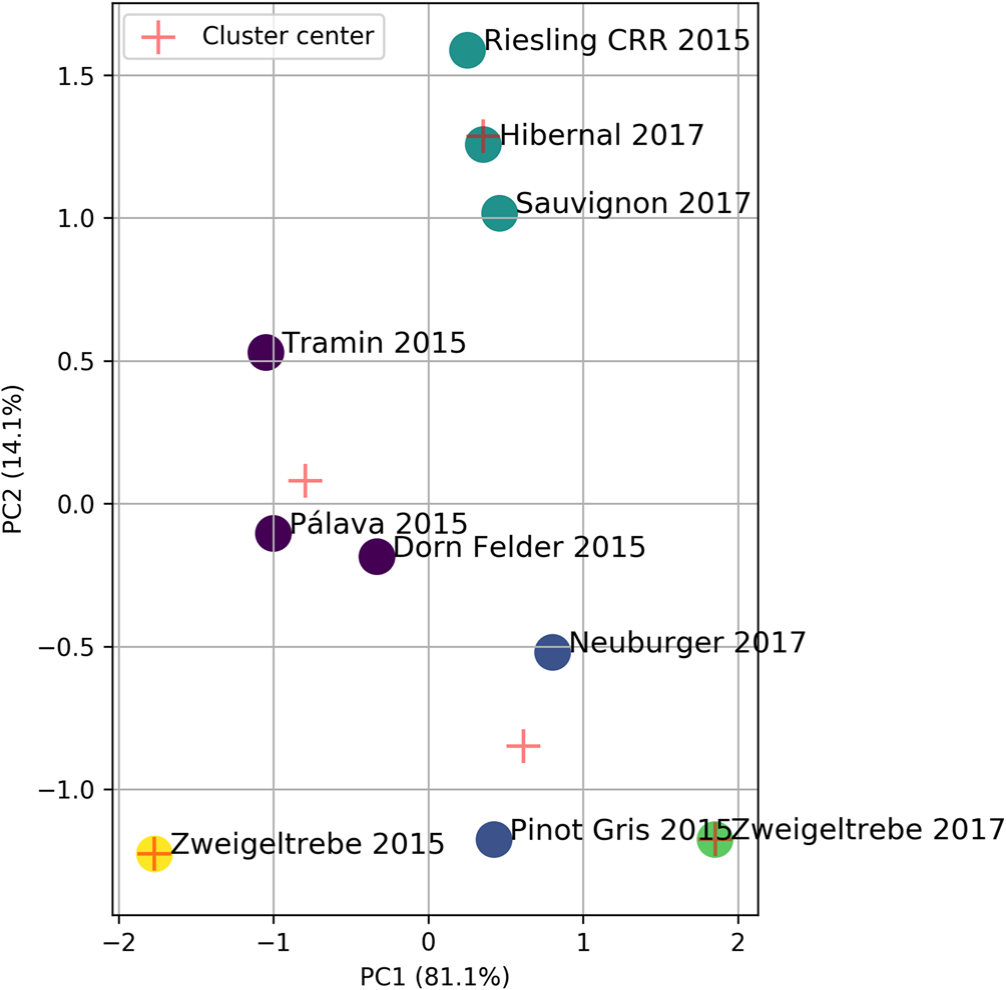
k-Means clusters for standardized PCs in analysis for data of FTIR spectra.

Next, based on the SSE criterion, five components were selected as the optimal choice for k-Means clustering for two normalized PCs reduced FTIR data. In this way we distinguish five clusters. The first one contains the oils Hibernal 2017, Riesling 2015, and Sauvignon 2017. The second one constitutes the next three types of oils, namely Dornfelder 2015, Pálava 2015 and Tramin 2015. The third contains Neuburger 2017 and Pinot Gris 2015 oils. The last two clusters are unit sets of oils Zweigeltrebe 2015 and Zweigeltrebe 2017, respectively. The clustering result is presented in Fig. 5.

#### GMM for clustering

**Figure 6:**
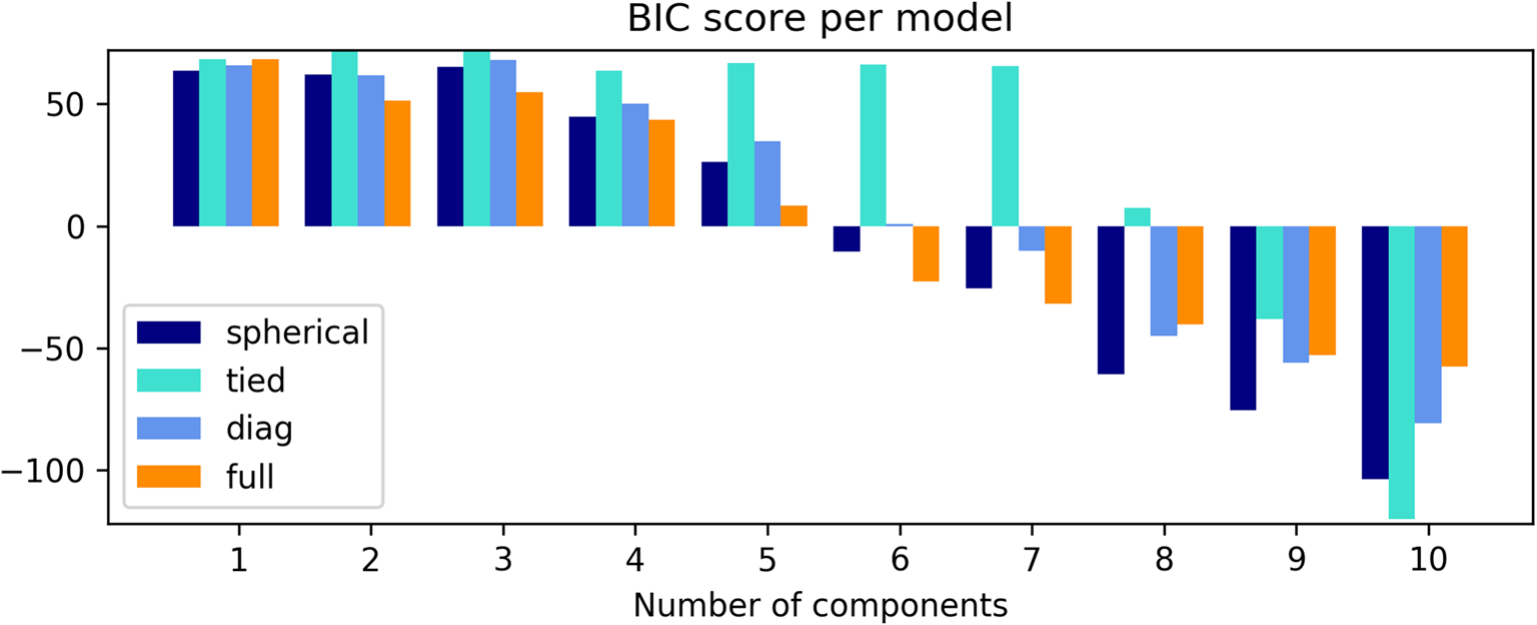
BIC score for various numbers of Gaussian components in GMM for data of FTIR spectra.

**Figure 7:**
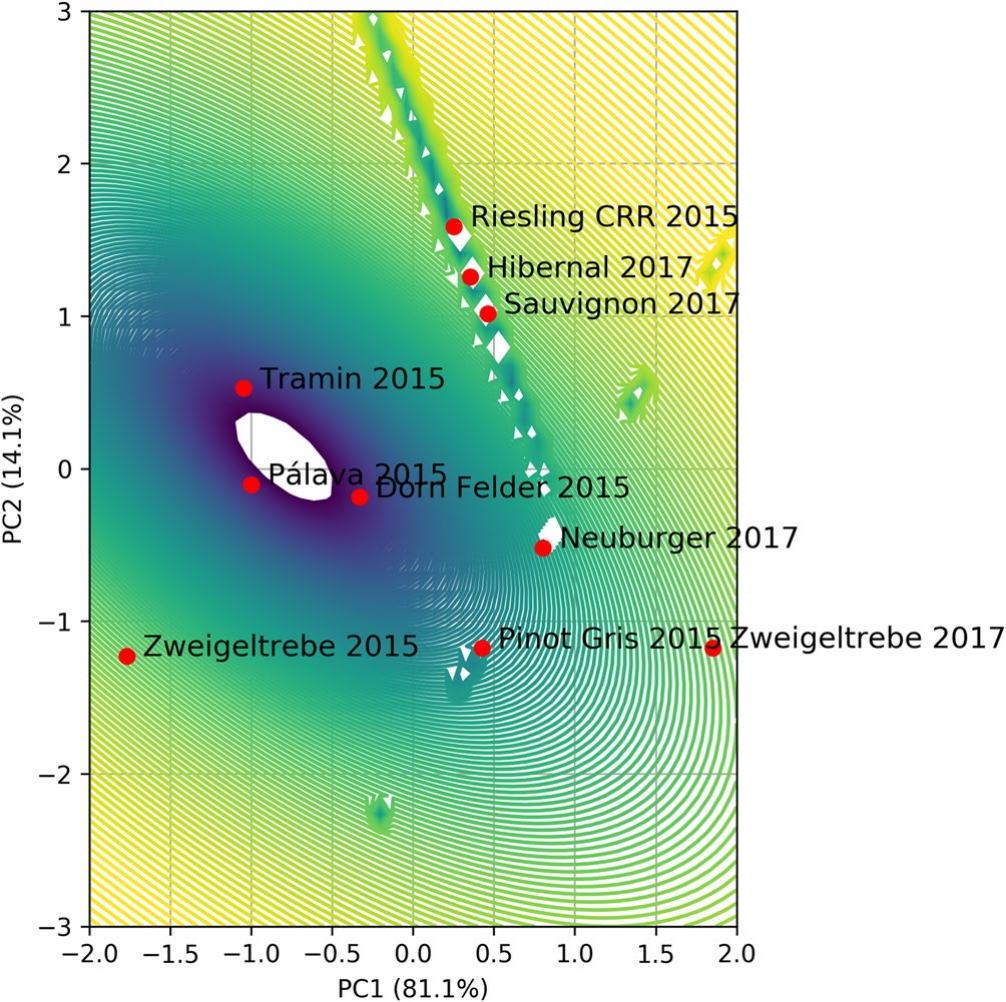
GMM for standardized 2PCs for data of FTIR spectra.

The parameters were extracted in GMM based on the number of clusters obtained with k-Means clustering. Estimation of the optimal model for Gaussians by five Gaussian components using BIC is presented in Fig. 6. Following the BIC criterion, the optimal Gaussian model for five components is ‘full’ (full covariance matrix). The values of the parameters from the fit are presented in Table 5 and the split into clusters is presented in Fig. 7.

**Table 5:** GMM parameters for standardized 2 PCs for data of FTIR spectra.

Description	Values
Mean	μ_1_ = [0.35639494, 1.2868471]
	μ_2_ = [1.85262144, − 1.17630937]
	μ_3_ = [− 0.793783, 0.07979547]
	μ_4_ = [0.61405915, − 0.84843964]
	μ_5_ = [− 1.76857353 − 1.22674042]
Weights	π_1_ = 0.3
	π_2_ = 0.1
	π_3_ = 0.3
	π_4_ = 0.2
	π_5_ = 0.1
Covariance matrices	$$\Sigma_{1} = \left[ {\begin{array}{*{20}l} {7.19461004 \times 10^{ - 3} } \hfill & { - 1.97590685 \times 10^{ - 2} } \hfill \\ { - 1.97590685 \times 10^{ - 2} } \hfill & {5.48141678 \times 10^{ - 2} } \hfill \\ \end{array} } \right]$$
	$$\Sigma_{2} = \left[ {\begin{array}{*{20}l} {1.0 \times 10^{ - 6} } \hfill & { - 1.12412679 \times 10^{ - 29} } \hfill \\ { - 1.12412679 \times 10^{ - 29} } \hfill & {1.0 \times 10^{ - 6} } \hfill \\ \end{array} } \right]$$
	$$\Sigma_{3} = \left[ {\begin{array}{*{20}l} {1.07131702 \times 10^{ - 1} } \hfill & { - 6.63095448 \times 10^{ - 2} } \hfill \\ { - 6.63095448 \times 10^{ - 2} } \hfill & {1.01725254 \times 10^{ - 1} } \hfill \\ \end{array} } \right]$$
	$$\Sigma_{4} = \left[ {\begin{array}{*{20}l} {3.57531835 \times 10^{ - 2} } \hfill & {6.20042065 \times 10^{ - 2} } \hfill \\ {6.20042065 \times 10^{ - 2} } \hfill & {1.07533499 \times 10^{ - 1} } \hfill \\ \end{array} } \right]$$
	$$\Sigma_{5} = \left[ {\begin{array}{*{20}l} {1.0 \times 10^{ - 6} } \hfill & {1.06496222 \times 10^{ - 29} } \hfill \\ {1.06496222 \times 10^{ - 29} } \hfill & {1.0 \times 10^{ - 6} } \hfill \\ \end{array} } \right]$$

## Conclusions

This study evidenced the efforts to characterize the selected grape oils in an unsupervised classification, based on their fatty acid composition and physical parameters, and FTIR spectroscopy. To this end, Gaussian Mixture Model based on Principal Component Analysis was applied. Two different approaches were compared. The first approach was based on fatty acids profile linked with physical parameters such as the apparent viscosity and mass density, while the second approach was based on the FT-IR spectroscopic data. The results obtained from these techniques help in the characterization and quality control of grape oils. In more detail, the fatty acids approach distinguishes the five clusters of considered grape oils. The first cluster contains each oil from the first class of FT-IR approach and one oil from the second FT-IR class (Riesling 2015, Tramin 2015, Hibernal 2017, Sauvignon 2017). The second approach consists of oils from the 2, 4 and 5 classes of FT-IR approach (Pálava 2015, Zweigeltrebe 2015, Zweigeltrebe 2017). The last clusters are created by oils from 3 and 2 classes of FT-IR approach. In general, the results obtained from both approaches are consistent and complementary with one another. The results of correlation analysis demonstrate that the concentration of MUFA is related to apparent viscosity.

In conclusion, the application of techniques associated with GMM-based clustering to classify features and characterize the grape oils may undoubtedly be considered as new tools to solve the characterization and clustering problems. Therefore, there is a promising prospect that methods used in this work will provide a basis suitable for addressing issues arising from the differentiation and unsupervised clustering in grape seed oils.

## Materials and methods

### Samples preparation

For the purpose of this work, the grape seed oils from 10 various grape types and 2 years (2015 and 2017) were used. In 2015 were included the varieties Dornfelder, Pálava, Pinot Gris, Riesling, Tramin, Zweigeltrebe and in 2017 the varieties Hibernal, Neuburger, Sauvignon, Zweigeltrebe. The relevant permission was obtained by the authors prior the samples harvesting from plants cultivated in South Morava, Czech Republic A prototype of a vibratory separator was used to separate the seeds from marc. For successful pressing of seeds and their storage, their initial moisture content was lowered to about 10% in a chamber dryer. The temperature in the chamber dryer did not exceed 40 °C. The material was kept in a closed bag at room temperature until screw pressing. All methods were performed in accordance with the relevant UE guidelines/regulations/legislation.

### Oil extraction from grape seeds

The oil was pressed on the screw press UNO FM 3F produced by the Farmet Company (Česká Skalice, CZ). This press model is designed for cold pressing of all oily seeds at 80 rpm. The pressing device components are: a matrix, 220 mm screw, head, heating mantle, nozzle holder, and nozzle in diameter 10 mm. After pressing, the oils were settled by gravity, then filtered, and poured into glass jars (volume 500 ml). Oils were not technologically treated or stabilized in any way.

### Physical properties

The density of oils was determined pycnometrically according to ISO 6883:2017^35^. The rheological evaluation of grape seed oils was prepared according to previously article^33^. The Rheometer Anton Paar MCR 102 (Graz, Austria) with the measuring geometry cone-plate was used. The gap between the cone and the plate was set at the stable value of 0.103 mm. The diameter of the cone equaled to 50 mm with the angle of 1°. Rheological tests were performed at the temperature 20 °C. The apparent viscosity was measured at the shear rate 5/s. Each physical properties analysis was performed in triplicate.

### Fatty acid profile

For our research we used the second part of ISO 12966 norm^36^, which specifies methods of preparing the methyl esters of fatty acids. Specifically, the boron trifluoride (BF3) transmethylation procedure was used. The isooctane solution thus obtained was prepared for analysis, by using the GC according to ISO 12966 norm, part four^37^. The profile of fatty acids was determined by using GC Hewlett Packard 4890D (Palo Alto, CA) with a flame ionization detector (FID). The separation was performed on column DB-23 (60 m × 0.25 mm with a 0.25 μm film thickness) from Agilent Technologies (Santa Clara, CA). The temperature program was as follows: the initial temperature was 100 °C held for 3 min, then was increased at 10 °C/min to 170 °C, then again increased at 4 °C/min to 230 °C held for 8 min, and then again at 5 °C/min to 250 °C held for 15 min. The injector temperature was 270 °C, while the detector temperature was set to 280°C. The injection volume was 2 µl at a split ratio of 40/1. The helium was used as a carrier gas with a flow rate of 1.0 ml/min. Retention times of FAME standards were used to identified individual fatty acid methyl esters. The resulting chromatograms were processed using the station CSW (version 1.7, Data Apex, Praha, CZ). Results are reported as % fatty acid (area under the peak of particular fatty acid) of total fatty acids (total area under the peak of all fatty acids). Each GC analysis was performed in triplicate. Chemicals used in the analysis were from VWR International (Radnor, Pennsylvania, USA) and FAME standards were from Supelco (Sigma-Aldrich, Saint-Louis, Missouri, USA).

### FT-IR measurements

Measurements of ATR-FTIR background-corrected spectra (25 scans for each sample) were carried out with the use of a HATR Ge trough (45° cut, yielding 10 internal reflections) crystal plate at 20 °C, and were recorded with a 670-IR spectrometer (Agilent, USA). The Ge crystal was cleaned with ultra-pure organic solvents (Sigma-Aldrich). The instrument was continuously purged with argon for 40 min. before and during measurements. Absorption spectra at a resolution of one data point per 1/cm (to the highest measurement accuracy) were obtained in the region between 4000 and 400/cm. Scans were Fourier-transformed and averaged with Grams/AI 8.0 software (Thermo Fisher Scientific, USA).

### Chemometric methods

The data were analyzed by correlations among variables were evaluated using principal component analysis (PCA), cluster analysis on normalized PCs (k-means and Gaussian Mixture Models <GMM>) to oils samples according to their acid and spectroscopy profile. The multivariate data analysis methods have found increased use during the last decades in all fields of spectroscopy-related research. Such methods are the state of the art of mathematical analysis. They perform a reduction of the dimensionality of data set and allows the visualization of underlying structure in experimental data and relationships between data and samples by identifying the directions in which most of the information is retained. The FTIR spectroscopy characterization of oils from grape seeds was combined with statistical analysis, PCA and GMM being considered as classification method of unsupervised learning. Characterization of the samples was performed using the relative intensity of absorption band corresponding to the main classes of chemical compounds identified in the IR spectrum was measured^21,22^. The spectral range was divide into fourth areas. The first spectral area, between 3050 and 4000/cm was not taken into account. It is known that this spectral range contains information that is not significant for oils discrimination (water absorbance) and it also can be derive of noise. The second spectral range 2605–3050/cm provided eight values of the absorption band intensity (every 50/cm) for analysis. The next spectral ranges, between 1600 and 2000/cm (every 50/cm) and between 700 and 1500/cm (every 50/cm) gave 9 and 17 values of the absorption band intensity for analysis, respectively^38^. Finally, we represented each IR spectrum as a vector with 34 values.

In order to discover underlying classes into which data of ten different oils set splits, the standard clustering methods from unsupervised learning were used in the following sequence.

1. Determine optimal Principal Components in PCA that explain above 70%.
2. Normalize PCs using standard scaler.
3. Using k-Means algorithm and the elbow rule to determine the optimal number of clusters into which normalized PC split.
4. Use the optimal number of clusters from the previous step to fix the number of Gaussian distributions in Gaussian Mixture Model (GMM) and determine optimal Gaussian parameters from the following^39,40^:
  - ‘full’—each component has its own covariance matrix;
  - ‘tied’—one general covariance matrix for each component;
  - ‘diag’—diagonal covariance matrices for each component;
  - ‘spherical’—each component has its own diagonal covariance matrix with equal eigenvalues;

The GMM has the following parametrization

$$p\left(x|\mu ,\Sigma \right)={\sum }_{k=1}^{n}{\pi }_{k}{p}_{k}\left(x|{\mu }_{k},{\Sigma }_{k}\right),$$

$${p}_{k}\left(x|{\mu }_{k},{\Sigma }_{k}\right)=\frac{1}{\sqrt{2\pi \left|\Sigma \right|}}exp\left(\frac{-1}{2}\left(x-{\mu }_{k}\right){\Sigma }_{k}{\left(x-{\mu }_{k}\right)}^{T}\right),$$

where π_k_ is the weight of the *k*-th Gaussian with normalization ${\sum }_{k=1}^{n}{\pi }_{k}=1$, μ_k_ is the mean of the Gaussian (its center), Σ_k_ is the covariance matrix.

The optimal is made by minimizing Bayesian Information Criteria (BIC)^38,41^ for a given number of components and models. The k-Means clustering as a way of choosing the optimal number of components prevents BIC selection to exclude one-cluster-per-point model. The trained model and the whole analysis pipeline can also be used for classifying new data. However, since the sample is small we did not do unsupervised machine learning here. In the analysis the version 0.20.0 of Scikit-Learn library^39,40^ was used.

## Data Availability

The samples of each material used in this study, namely the variety of grape seed oils are available on request from M.V and P.B laboratories.
